# Association of parental-reported vitamin D supplementation with dental caries of 3-year-old children in Poland: a cross-sectional study

**DOI:** 10.1007/s00784-021-03914-8

**Published:** 2021-04-08

**Authors:** Dorota Olczak-Kowalczyk, Urszula Kaczmarek, Dariusz Gozdowski, Anna Turska-Szybka

**Affiliations:** 1 Department of Paediatric Dentistry, Medical University of Warsaw, Binieckiego St. 6, 02-097 Warsaw, Poland; 2 Department of Conservative Dentistry and Paedodontics, Medical University of Wroclaw, Krakowska St 26, 50 – 425 Wrocław, Poland; 3 Department of Experimental Design and Bioinformatics, Department of Agriculture and Biology, Warsaw University of Life Sciences, Nowoursynowska 166 ST., 02-787 Warsaw, Poland

**Keywords:** Caries, Early childhood caries (ECC), Parental-reported vitamin D supplementation, Preschool children, Severe early childhood caries (S-ECC)

## Abstract

**Objective:**

The study aimed to assess the association between parental-reported vitamin D supplementation and caries in a national sample of 3-year-olds in Poland.

**Materials and methods:**

A total of 1900 children, representing all provinces of Poland, were invited. The questionnaires concerned vitamin D supplementation, socio-demographics, and oral health behaviours. Based on dental examination, caries scores (dmft/dmfs), prevalence of early childhood caries (ECC) and severe ECC (S-ECC) were calculated. The Spearman’s correlation, linear regression and logistic regression were used to assess the association between various factors and caries (*p* < 0.05).

**Results:**

A total of 1638 children were tested. Of this number, 99.1% infants were supplemented with vitamin D. Supplementation had been continued seasonally in 55.2% children. ECC/S-ECC prevalence were significantly lower in children receiving vitamin D (ECC 38.3% vs. 44.7%, OR = 0.77; S-ECC 20.5% vs. 27.1%, OR = 0.69; *p* < 0.05). Mean dmft/dmfs were lower in those with supplementation (1.54 ± 2.72 vs. 2.24 ± 3.55; 2.40 ± 5.56 vs. 3.72 ± 7.56, respectively; *p* < 0.001). After controlling for confounding factors, supplementation was not significantly associated with caries; only dt/ds were still associated. Maternal education, sweetened beverages before bedtime, bottle use were significantly associated with S-ECC.

**Conclusions:**

Lower caries prevalence was observed in those with vitamin D supplementation. The association between parental-reported vitamin D and ECC/S-ECC was not significant in Polish children. Decayed teeth and supplementation were still associated. Dietary habits can modify the association with caries.

**Clinical relevance:**

There may be an association between vitamin D supplementation and lower caries in children. Parents should supplement their children during periods of significant growth and development.

## Introduction

Early childhood caries (ECC) and severe ECC (S-ECC) represent a significant public health challenge in many countries [1, 2]. They often remain untreated and have an important impact on oral and general health, on body’s growth and development and on the quality of life of families and communities [3]. In Europe, ECC levels vary [4, 5]. In Western Europe, it is at 11.7–14.5% in 3-year-olds and at 31–41% in 5-year-olds [4, 5]. In Poland, the figures for 3-year-olds and 5-year-olds are 41.1% and 76.8%, respectively [6, 7]. ECC has a number of causes and develops as a reaction to a combination of various socio-demographic and behavioural factors [8]. Some studies have shown an association of low vitamin D levels and dental caries in children, while others have not shown any correlation or the association [4, 5, 9–22]. There is clinical evidence of the association of vitamin D deficiency with dental caries in the primary dentition among Canadian populations; they are at risk of vitamin D deficiency due to high latitude geographical location [16]. Based on systematic review and meta-analysis Hujoel [12] concluded that vitamin D had topical fluoride–like characteristics.

Several possible mechanisms have been proposed to explain the role of vitamin D in reducing caries risk, such as the vital role of vitamin D in regulating serum calcium, phosphate and parathyroid hormone levels. Calcium and phosphate homeostasis is essential for the formation, calcification, mineralisation and health of hard tissues like oral bone and teeth [9, 12, 14, 19, 22]. Primary maxillary anterior teeth begin to calcify during the second trimester (~16 weeks) and continue to do so until three months postnatally, as do other primary teeth [14]. Studies on the molecular basis of vitamin D and vitamin D receptor (VDR) have concluded that vitamin D influences tooth germ formation; it contributes to the regulation of enamel and dentine formation and maturation, and controls the stages of tooth crown development [22]. In addition, vitamin induces the formation of certain antimicrobial peptides (AMPs) such as defensins and cathelicidins, which are relevant elements in breaking the membrane integrity of the oral bacteria [15, 22, 23]. Salivary antimicrobial peptides concentrations showed large differences between individuals, with a significantly higher level of salivary defences in caries-free children. Vitamin D prevents infection by regulating B-cell proliferation and immunoglobulin production.

The secretion rate and saliva quality play a role in caries development and also in remineralization [23]. Vitamin D is essential for the maintenance and utilisation of a specific pool of calcium required for normal fluid and electrolyte of the saliva of the parotid gland. Various caries-protective factors, such as calcium, inorganic phosphate, pH-increasing substances and antimicrobial agents, are present in saliva. Hence, vitamin D deficiency is an important environmental factor in predisposition to dental caries [4, 5, 9–22].

Vitamin D is an important prohormone that can be synthesised by the skin exposed to sunlight (UVB) or ingested with food. The ever-present vitamin D deficiency is a global public health problem [4, 9, 24]. Poland is also at high risk of vitamin D deficiencies because of its geographical position and, therefore, insufficient sun exposure. Additionally, limited outdoor activity, sun protection, unhealthy diet poor in vitamin D and low vitamin D content of staple foods can compound this health issue [25, 26]. The concentration of 25 (OH)D can be increased by changing lifestyles and taking vitamin D supplements. Vitamin D supplementation in Poland is based on actual recommendations known as “Reference standard”, which should be introduced from the first days of life: supplementation of 400 international units (IU)/day up to the age of 6 months and supplementation of 400–600 IU/day between 6^th^ and 12^th^ month of age. In children and adolescents (1–18-year-olds) supplementation of 600–1,000 IU/day is recommended between September and April [25, 26]. A paediatrician in a public health care facility is responsible for introducing adequate prophylactic measures. Their duties include the selection of an appropriate preparation allowing for patient’s specific needs and financial means of their family. The drops are not refunded by the state, which may result in a limited access to vitamin D supplements. In Central Europe, the weather conditions favouring vitamin D synthesis occur between late April and early September [25, 26]. Regular vitamin D supplementation is the most effective way to obtain its proper supply whenever exposure to sunlight is insufficient. Improvement in the diet will still be responsible for mere 20% of the required daily supply, and is insufficient to obtain the target concentration of 25(OH)D [25, 26]. From this perspective, the population of children—being the most susceptible to short- and long-term consequences of vitamin D deficiency—should be beneficiary to state-financed vitamin D supplementation [26]. Even though the association between vitamin D supplementation and dental caries in children has been reported [4, 5, 13, 14, 16–19], few studies have been conducted in Poland, and those which have been, concerned smaller samples and different age groups [27, 28].

The aim of the study was to assess the possible association between parental-reported vitamin D supplementation and dental caries on a national sample in the paediatric population of 3-year-olds living in all sixteen provinces of Poland.

The null hypothesis was that caries experience should be statistically significantly lower in children receiving parental-reported vitamin D supplementation in comparison with children who were not provided with such supplementation. The outcome variable was the presence of dental caries scores (dmft/dmfs indices).

## Material and methods

The study was performed as a cross-sectional national survey from October to November 2017. In all 16 provinces of Poland, administrative divisions of second level (counties) and third level (communes that are classified as urban or rural) were randomly chosen. The population study group was defined by a three-stage cluster sampling procedure: selection of states (the first large cluster), then selection of samples of kindergartens (second-level cluster), followed by samples of groups (third-level cluster) and finally samples of 3-year-old children. In the next stage, 137 preschools, both public and private, were randomly selected. Data on the total number of 3-year-olds were derived from the Central Statistical Office [29].

### Study population

The sample size was calculated basing on data concerning caries prevalence in this age group, i.e. about 50% caries-free subjects. With such an assumption in mind, and a 95% level of confidence and ± 2.5% error tolerance, 1514 subjects represented a minimum sample size. A representative study sample was recruited based on WHO criteria [30]. The number of invited participants exceeded the minimum sample size by 25%, accounting for potential refusal to participate at the clinical stage. Requests for signed consents from the child’s parent along with a letter informing about the scope of the survey and a questionnaire were distributed by the preschool teacher to the parent, who was supposed to complete the questionnaire at home and return it.

The inclusion criteria were as follows: a child attending the preschool at the age of 3 years, present at the time of the study in the preschool and subjected to oral examination following submission of the written consent of a parent/caregiver and a fully answered questionnaire. The exclusion criteria were: the child at the age of less than 3 years and over 4 years, individuals with diagnosed vitamin D deficiency requiring higher doses of vitamin D than those recommended for the general population, children who did not comply with screening protocols, or absent on the day of the examination, no written consent from a parent/caregiver and incompletely filled questionnaire. Patients who had had previous chronic disease or those undergoing any drug therapy were excluded.

### Clinical examination

The dental examinations were conducted in artificial light using plane mouth mirrors and metallic metal periodontal probes (Community Periodontal Index (CPI) probe) that conform to WHO specifications (0.5 mm ball tip). These examinations were performed in accordance with the WHO standard criteria and procedures for epidemiological surveys [30]. Children were examined in an upright position in a chair with a high backrest with the examiner standing in front of the child. Immediately before the oral examination, the children were instructed by dentists to brush their teeth. The examiners dried the surface of the teeth with cotton rolls and swabs. The presence of carious lesions was assessed at both the tooth and the surface levels. After the examination, a report was given to the child’s parents/caregivers to inform them if the child needed treatment. Decayed, missing and filled teeth and surfaces were assessed according to the World Health Organization Oral Health Survey guidelines criteria [30]. The prevalence of ECC (the presence of one or more decayed (non-cavitated or cavitated lesions), missing (due to caries) or filled tooth surfaces in any primary tooth) and S-ECC (any sign of smooth-surface caries in a child younger than 3 years of age, and from ages three through five, one or more cavitated, missing (due to caries), or filled smooth surfaces in primary maxillary anterior teeth or a decayed, missing, or filled score of greater than, or equal, four (age 3)) as well as mean dmft and dmfs was calculated [31]. The dental examinations were conducted by 16 teams (from each province of Poland) consisting of two dentists trained in survey methodology, specialists in paediatric dentistry, with many years of experience.

Training and calibration of the examiners was conducted according to the WHO recommendations, so that the examiners shared similar clinical reliability, which was additionally verified by another 10% sample randomly selected as control examination [30]. The assessment of the consistency of each individual examiner (intra-examiner reproducibility) and the variations between examiners (inter-examiner reproducibility) were performed. Each paediatric dentist (examiner) independently examined the same group of ten patients, and the findings were compared with those of the experienced supervisor. Cohen’s kappa coefficient between the reference dentist (AT-S) and the other dentists was between 0.857 and 1.000 for carious teeth, and should be interpreted as an almost perfect agreement.

### The questionnaire

The questionnaire included demographic and social background (gender, area of residence, mother’s age, education and the economic status, child’s oral health–related practices, e.g. frequency of tooth brushing, adult-supervised tooth brushing, the use of fluoride toothpaste, eating habits (sweetened beverages before bedtime and at night, frequency of consumption of sweetened sodas/beverages, milk consumption, bottle-feeding) and parental-reported vitamin D supplementation (whether it was used in the first year of life, and whether vitamin D is presently received at least in autumn and winter months, following Polish recommendations), fluoride prophylaxis, and dental check-ups).

Analysis of the socio-economic status (SES) included two of three variables: mother’s education and the economic status, but disregarded occupation. In Poland, legally determined 5-level classification of education include: primary (elementary), vocational, secondary, post-secondary and higher (https://pl.wikipedia.org/wiki/Wykształcenie; https://en.wikipedia.org/wiki/Education). The authors decided to form three categories in the following fashion: 1 – primary and vocational, 2 – secondary and post-secondary, and 3 – higher. The respondents failed to reveal their monthly household income, and so the economic status criterion was subdivided into three levels (high, middle and low) depending on one variable—respondent’s subjective evaluation (https://en.wikipedia.org/wiki/Socioeconomic_status).

The questionnaire was designed by the principal investigator (DO-K) and based on the previously used questionnaires in Polish national oral health surveys as part of “Monitoring of oral health of the population of Poland”, which has been continued since 1999. The programme follows the guidelines set by the WHO, which recommends the use of simplified structured questionnaires for the collection of data on oral health and caries risk factors in children; however, the questions were adapted to national specificity and included for the first time, among others, additional questions concerning vitamin D [30]. The questionnaire was not based strictly on the literature concerning vitamin D questionnaires (VDQ) to quantify the intake of vitamin D through diet and sun exposure.

The recommended vitamin D supplementation was the one factor that determined the 25(OH)D status. The authors did not account for potential modifiers of the 25(OH)D status, such as the exposure to sunlight or intake of foodstuffs containing vitamin D or vitamin D–fortified foods. The survey aimed to estimate actual vitamin D supplementation, at least in autumn and winter seasons, as recommended in Poland. There were no questions regarding pregnancy (prenatal vitamin D/multivitamin use) or vitamin D-related nutrition. In childhood, intake from supplements, rather than from UV light exposure or fortified foodstuffs, is likely to be the major determinant of vitamin D status in Poland, similarly to other geographical regions at risk of deficiencies. In this study, the authors were able to internally validate the questionnaire findings because participants had undergone clinical dental examinations. Blood samples for serum 25(OH)D were not collected from children.

### Statistical analysis

Parental-reported vitamin D supplementation in all the analyses was treated as a binomial variable (0 – lack of supplementation, 1 – with supplementation). The assessed variables were presented as percentages (with 95% confidence intervals, CI) or mean values with standard deviation (SD).

Educational status of the mother was used as a binomial variable (higher education – 1 vs. others – 0) for logistic regression or as a discrete variable with five levels (primary – 1 to higher education – 5) for multiple linear regression. Economic status for multiple linear regression was used as a discrete variable with three levels (1 – low to 3 – high).

Percentages of subjects who used vitamin D supplementation were compared between levels of various factors using chi-squared test. The Spearman’s rank correlation coefficient was used to assess the association between various factors and caries prevalence in children. The simultaneous association between various variables and the dmft index was evaluated using multiple regression. Furthermore, a simple logistic regression (with calculation of odd ratios, OR) was performed for dichotomous variables describing caries severity, separately including the association with each factor calculated individually, and a multiple logistic regression (with calculation of adjusted odd ratios, AOR) (with Wald’s test) to assess the simultaneous association with several factors. In simple logistic regression, the only independent variable was parental-reported vitamin D supplementation (with supplementation vs. without supplementation), and the dependent binomial variables were dmft (equal 0 vs. greater than 0), ECC (present vs. not present), S-ECC (present vs. not present). Moreover, three different multiple logistic regression models were applied with various sets of confounders: (1) parental-reported vitamin D supplementation (with supplementation vs. without supplementation), and teeth brushed at least twice a day vs. others; (2) parental-reported vitamin D supplementation (with supplementation vs. without supplementation), teeth brushed at least twice a day vs. others, mother’s education (higher education vs. others), sweetened beverages (before bedtime and at night vs. others) and sweetened sodas/beverages (once a day or more often vs. others); (3) the same confounders as for model (2) and additionally bottle-feeding vs. no bottle-feeding. OR and AOR together with confidence interval (CI at 95% confidence level) basing on logistic regression were calculated. The confounders at AOR calculations included factors statistically significantly correlated with caries. Furthermore, Mann–Whitney *U* test was used to determine the significance of the differences between means for the two groups. SPSS 22, Statistica 10 and R 3.2 were used for statistical analysis. Significance level for all the analyses was set at 0.05.

## Results

The participation in the study was voluntary. Approval from the selected 127 preschool authorities, 63 in urban and 64 in rural areas, was obtained. A total of 1900 children were invited to participate in the study; however, 4.8% (*n* = 92) of the parents/caregivers did not respond to the request for granting permission and 1.1% (*n* = 22) did not complete the questionnaire. Additionally, 2.5% (*n* = 48) of the sampled children were absent at the time of dental examination and 5.3% (*n* = 100) refused to be examined. Eventually, 1638 children were included in the study (exceeding a minimum representative sample size). The socio-demographics, the oral health-related behaviours and parental-reported vitamin D supplementation appear in Table 1.

**Table 1:** The socio-demographics, the oral health-related behaviours and parental-reported vitamin D supplementation

Parameters		*N*/% (95% confidence interval)
Socio-demographics factors		
Number of patients		1638/100%
Child’s gender	Female	859/52.4% (49.0–56.1)
	Male	779/47.6% (44.3–51.0)
Mean mother’s age ± SD (years)		29.2 ± 4.84
Place of residence	Urban area	866/52.9% (49.4–56.5)
	Rural area	772/47.1% (43.9–50.6)
Mother’s education	Primary/vocational	163/10.0% (8.4–11.6)
	Secondary/post-secondary	536/32.7% (30.0–35.6)
	Higher	939/57.3% (53.7–61.1)
Economic status	Low	192/11.7% (10.1-13.5)
	Average	970/59.2% (55.6–63.1)
	High	476/29.1% (26.5–31.8)
Average number of children in the family ± SD		1.86 ± 0.79
Oral health–related behaviours		
Dental check-up in the past 6 months		573/35.0% (32.2–38.0)
Application of fluoride varnish		176/10.7% (9.2–12.5)
Teeth brushed at least twice a day		868/53.0% (49.5–56.6)
Adult-supervised tooth brushing		566/38.9% (35.8-42.2)
For children aged between 1 and 2 years serving	Sugar-sweetened foods	1420/86.7% (82.2–91.3)
	Sweetened beverages before bedtime and at night	278/17.0% (15.0–19.1)
Serving once a day or more often	Sweetened sodas	45/2.8% (2.0–3.7)
	Fruit juices	561/34.2% (31.5–37.2)
	Sweets and bars	144/8.8% (7.4–10.4)
	Biscuits, cupcakes, cakes	206/12.6% (10.9–14.4)
Parental-reported vitamin D supplementation		
	In the first year of life	1623/99.1% (94.3–100.0)
	Presently receiving vitamin D at least in autumn and winter months	904/55.2% (51.7–58.9)

Each of the 16 provinces of Poland was represented by at least 100 children living in cities and neighbouring rural areas (Table 2).

**Table 2:** The number and percentages of patients included in the study living in different provinces of Poland presently receiving supplementation of vitamin D

	Provinces (Polish names)	Cities*	Total number of patients	Number (percentages) of patients presently receiving vitamin D supplementation
1.	wielkopolskie	Krotoszyn	100	80 (80.0%)
2.	kujawsko-pomorskie	Nakło	101	50 (49.5%)
3.	małopolskie	Tarnów	106	70 (66.0%)
4.	łódzkie	Wieluń	101	49 (48.5%)
5.	dolnośląskie	Oława	100	56 (56.0%)
6.	lubelskie	Lublin and Włodawa	100	45 (45.0%)
7.	lubuskie	Żary	100	39 (39.0%)
8.	mazowieckie	Radom	100	55 (55.0%)
9.	opolskie	Krapkowice	111	53 (47.7%)
10.	podlaskie	Suwałki	100	68 (68.0%)
11.	pomorskie	Wejherowo	103	74 (71.8%)
12.	śląskie	Jastrzębie-Zdrój	100	56 (56.0%)
13.	podkarpackie	Jasło	100	52 (52.0%)
14.	świętokrzyskie	Starachowice	101	44 (43.6%)
15.	warmińsko-mazurskie	Elbląg	100	62 (62.0%)
16.	zachodniopomorskie	Szczecin and Świdwin	115	51 (44.3%)

*Cities with neighbouring rural area included in the study

Dental examinations revealed that 674 (41.2%) of children manifested ECC including 38.3% children (346/904) who received vitamin D supplementation and 44.7% (328/734) without supplementation. Considering S-ECC, its prevalence was observed in 384 children (23.4%), in 20.5% (185/904) with supplementation and 27.1% (199/734) without it. The differences were statistically significant (ECC *p* = 0.018, S-ECC *p* = 0.002).

Mean dmft and dmfs levels significantly differed between those supplemented and those that were not (1.85 ± 3.14 and 2.99 ± 6.56, respectively) (Table 3).

**Table 3:** Mean values of dmft/dmfs scores and their components as related to parental-reported vitamin D supplementation

Parameters	Parental-reported vitamin D supplementation		*p* based on Mann–Whitney *U* test
	Yes	No	
	Mean ± SD		
dmft	1.54 ± 2.72	2.24 ± 3.55	<0.001*
dt	1.43 ± 2.62	2.03 ± 3.33	0.003*
mt	0.01 ± 0.13	0.02 ± 0.47	0.971
ft	0.10 ± 0.48	0.18 ± 0.86	0.582
dmfs	2.40 ± 5.56	3.72 ± 7.56	0.001*
ds	2.27 ± 5.42	3.41 ± 7.10	0.004*
ms	0.01 ± 0.21	0.11 ± 2.09	0.971
fs	0.12 ± 0.61	0.20 ± 1.05	0.589

*Mann–Whitney *U* test; *p* < 0.05

*dmft*, decayed, missing, filled primary teeth; *dmfs*, decayed, missing, filled surfaces; *dt(s)*, decayed tooth (surface); *mt(s)* missing tooth (surface); *ft(s)*, filled tooth (surface); *SD*, standard deviation

A total of 566 (38.9%) children had their teeth brushed by an adult. Fluoridated toothpaste was used by 1158 (70.7%) of children. There were no children receiving fluoride systemically through drinking water. Community drinking water is not fluoridated in Poland; the level of fluoride in drinking water was <0.5 mg F/l. Only 176 (10.7%) of children had fluoride varnish applied by a health professional. Milk was consumed at the time of examination by 478 (29.2%) of children, and 1327 (81.1%) were bottle-fed.

The *Spearman correlation* showed that caries was associated with current parental-reported vitamin D supplementation (*R* = − 0.083), the mother's education level (*R* = −0.149), brushing teeth at least twice a day (*R* = −0.063), serving sugar-sweetened beverages before going to bed and at night to children aged 12 months and younger (*R* = 0.135), the number of children per household (*R* = −0.103, *p* < 0.001). Caries was also correlated with the economic status of the family (*R* = -0.70) and with other improper dietary habits, especially with drinking sweetened sodas at least once a day (R = 0.105). Having more children in one family reduced the probability of children presently receiving vitamin D (*R* = -0.103, *p* < 0.001). The Spearman’s rank correlation coefficient did not confirm any association between parental-reported vitamin D supplementation in infants and dental caries.

Parental-reported vitamin D seasonal supplementation in autumn and winter seasons (following Polish recommendations) more frequently concerned children of mothers with high education and those in the 25 and > 25 years age group) (Table 4). Supplementation in all the analyses was treated as a binomial variable (0 – lack of supplementation, 1 – with supplementation).

**Table 4:** Presently receiving parental-reported vitamin D supplementation depending on the place of residence, mother’s education and age and the economic status

Presently receiving parental-reported vitamin D supplementation		*N*/% (95% confidence interval)	*p*
Total		904/55.2% (51.7–58.9)	
Place of residence	Urban area	496/57.3% (52.4–62.5)	0.072
	Rural area	408/52.8% (47.8–58.2)	
Mother’s education	Primary/vocational	68/36.0% (27.9–45.6)	<0.001*
	Secondary/post-secondary	257/47.9% (42.2–54.1)	
	Higher	579/61.7% (56.8–67.0)	
Mother’s age	<25 years of age	114/47.9% (39.5–57.5)	0.047*
	25-35 years of age	635/56.6% (52.3–61.2)	
	>35 years of age	92/56.1% (45.2–68.8)	
Economic status of the family	Low	103/53.6% (43.8–65.1)	0.870
	Average	535/55.1% (50.5–60.0)	
	High	266/55.9% (49.4–63.0)	

*chi-square test; *p* < 0.05

Results of multiple linear regression show a significant (*p* < 0.05) association of parental-reported vitamin D supplementation with the dmft score (Table 5). Supplementation was observed to have a positive association, i.e. decreasing the dmft score in the studied group. Another significant association (*p* < 0.05) was observed for the place of residence (higher dmft scores in villages), educational level of the mother (higher education was related to lower dmft), and the economic status (higher economic status was related to lower dmft).

**Table 5:** Multiple linear regression of the association between different independent variables and dmft as a dependent variable

	Dependent variable: dmft	
Independent variables	Standardised regression coefficient	*p*
Parental-reported supplementation of vitamin D	−0.083	0.001*
Place of residence (city – 0/village – 1)	0.048	0.049*
Education level of mother (from primary – 1 to higher – 5)	−0.168	<0.001*
Mother’s age (at childbirth)	−0.022	0.364
Economic status (three levels, from low – 1 to high – 3)	−0.066	0.007*

*Significant association at 0.05 significance level

Unadjusted odd ratios (ORs) and adjusted AORs, which controlled for confounders like oral health behaviours (dietary habits and tooth brushing), also showed lower likelihood of caries prevalence in the group receiving vitamin D supplementation (Table 6). However, positive association with vitamin D supplementation was not significant if several confounders were included in the logistic regression model (*p* > 0.05 for AOR-2 and AOR-3). Maternal education, sweetened beverages before bedtime, bottle use were significantly associated with S-ECC (AOR-3: 0.57, *p* < 0.001; 1.87, *p* < 0.001; 0.66, *p* = 0.005, respectively). Higher education may increase awareness of a variety of pro-health behaviours, and in this way may have an indirect association with ECC/S-ECC. At the same time, consumption of sweetened beverages at bedtime or at night had an adverse association, considerably contributing to increased severity of ECC and S-ECC. Also bottle-feeding had a negative association, but only with S-ECC. The following variables were excluded from the multiple logistic regression model because their association did not significantly affect ECC or S-ESS: milk at least once a day vs. others (*p* = 0.852 and *p* = 0.358), the use of fluoridated toothpaste vs. non-fluoridated toothpastes (*p* = 0.603 and *p* = 0.872), low and medium economic status vs. high (*p* = 0.297 and *p* = 0.331) and the place of residence (city vs. village) (*p* = 0.236 and *p* = 0.087). Values in brackets present *p*-values based on multiple logistic regression for ECC and S-ECC as the dependent variable, respectively.

**Table 6:** Simple and multiple logistic regression for the prevalence of caries depending on parental-reported vitamin D supplementation and association between vitamin D and other factors on ECC and S-ECC

Parameters	Odds ratios (OR) and adjusted odds ratios (AOR)							
	OR (95% CI)	*p*	AOR-1 (95% CI)	*p*	AOR-2 (95% CI)	*p*	AOR-3 (95% CI)	*p*
Dependent variable: ECC								
Parental-reported vitamin D supplementation	0.77 (0.63–0.94)	0.009*	0.81 (0.67-1.00)	0.048*	0.85 (0.69–1.04)	0.128	0.86 (0.70–1.05)	0.136
Teeth brushed at least twice a day vs. others			0.83 (0.68–1.01)	0.061	0.87 (0.71-1.07)	0.185	0.87 (0.71–1.06)	0.168
Mother’s higher education vs. others					0.70 (0.57–0.86)	<0.001*	0.69 (0.57–0.85)	<0.001*
Sweetened beverages before bedtime and at night vs. others					1.60 (1.23–2.08)	0.001*	1.61 (1.24–2.10)	<0.001*
Sweetened sodas/beverages once a day or more often vs. others					1.42 (0.77–2.61)	0.262	1.44 (0.78–2.65)	0.244
Bottle-feeding vs. no bottle-feeding							0.83 (0.64–1.07)	0.144
Dependent variable: S-ECC								
Parental-reported vitamin D supplementation	0.69 (0.55–0.87)	0.002*	0.75 (0.59–0.95)	0.015*	0.80 (0.63–1.01)	0.065	0.81 (0.64–1.02)	0.076
Teeth brushed at least twice a day vs. others			0.80 (0.64–1.01)	0.065	0.87 (0.67–1.10)	0.239	0.86 (0.68–1.09)	0.201
Mother’s higher education vs. others					0.59 (0.47–0.75)	<0.001*	0.57 (0.45–0.73)	<0.001*
Sweetened beverages before bedtime and at night vs. others					1.81 (1.37–2.42)	<0.001*	1.87 (1.40-2.48)	<0.001*
Sweetened sodas/beverages once a day or more often vs. others					1.45 (0.77–2.74)	0.255	1.50 (0.79–2.84)	0.210
Bottle-feeding vs. no bottle-feeding							0.66 (0.50–0.88)	0.005*

*Significant association based on Wald test; *p* < 0.05; *OR*, odds ratio based on simple logistic regression; *AOR*, adjusted odds ratio based on multiple logistic regression; *AOR1*, adjusted odds ratio for the first multivariate model where confounder is oral health-related behaviours (teeth brushed at least twice a day vs. others); *AOR2 confounders*, oral health-related behaviours (teeth brushed at least twice a day vs. others), mother’s education level (higher education vs. others) and consumption of sweetened beverages (before bedtime and at night vs. others and once a day or more often vs. others). *AOR3 confounders*, the same as for *AOR2 *and additionally bottle-feeding vs. no bottle-feeding

## Discussion

To the best of the authors’ knowledge, this study is the first one to present a comprehensive view on the possible association of parental-reported vitamin D supplementation with dental caries on a nationally representative sample of 3-year-olds living in all sixteen provinces of Poland. The study was conducted as part of the Ministry of Health national programme—the only nationwide programme assessing oral health and causative factors of oral diseases. Even though the programme was launched in 1999, it was in 2017 that it was extended with an additional factor of vitamin D supplementation. Similar studies had been carried out in Poland but on a local scale and on smaller samples [27, 28]. Reliable data or studies on larger populations are not available.

One of the important findings of the present study was that as many as nearly 100% of the families followed the recommendation to supply children with vitamin D in the first year of their life, but only half of 3-year-olds continued supplementation despite recommendations [25, 26]. Many infants and children >5 years of age may not receive the supplementation, in spite of growing awareness [24, 32–35]. In Australia and in Canada children are reported to have been taking supplements on a daily basis in 10% and 29.45%, respectively [10, 11, 36]. A much higher intake was reported in the Germany and Netherlands (91%), likewise in the present study [32, 35]. According to the results of the present study—the older the mother with higher level of education, the more frequent vitamin D supplementation. Contrary, Dratva et al. [37] indicated that mothers aged over 35 years, as well as <25 years, gave vitamin D less often. No evidence could be found confirming the association with the economic status, similarly to the study by Munasinghe et al. [11] and contrary to those by Schroth et al. [9] and Black et al. [10].

The primary goal of the present study was to assess the association between parental-reported vitamin D supplementation and dental caries in 3-year-old children who had been supplemented in infancy. Although the authors did not find this association after controlling for many confounding variables, the association was significant for one component of the dmft/dmfs indices: dt and ds. The obtained results make it possible to formulate a thesis that children with carious lesions (dt/ds) have not been supplemented with vitamin D. These findings may be in some way consistent with those reported by Schroth et al. [38], in which significance was seen in an inverse relation between cord 25(OH)D levels and dt scores; however, this was not sustained on regression modelling. Results similar to those obtained in the present study have been presented in the cohort Iowa Fluoride Study indicating an association between inadequate (low) intake of vitamin D with increased caries experience [39]. Brown et al. [4] confirmed that a high proportion of children aged below 5 years, presenting with dental caries, were deficient in vitamin D.

Some studies have shown an association of low vitamin D levels and dental caries, while others have not shown any correlation or the association [4, 5, 13, 14, 16–19, 28, 37, 40]. Schroth et al. [9, 16, 17] described the significant inverse correlation between 25(OH)D levels and caries scores. Hujoel [12] stated that “vitamin D exposures in early life may play a role in caries prevention” [12]. Supplemental vitamin D was associated with a significant reduction in the incidence of dental caries compared with no supplementation (RR 0.53, 95% CI 0.43 to 0.65; 38) [12]. Hujoel [12] also concluded that vitamin D has topical fluoride-like characteristics. In the currently examined group of children, the absence of vitamin D supplementation was not the only unfavourable behaviour. Only half of the examined children had their teeth brushed twice a day. Most children were given sugary foodstuffs. Once hygiene and eating habits were included in the confounders, AORs for the association between supplementation of vitamin D and ECC and S-ECC was slightly higher. The adverse association between consumption of sweetened beverages and increased prevalence of ECC (AOR-2 = 1.60) and S-ECC (AOR-2 = 1.81) was confirmed, while the association with hygienic behaviour is not significant. Fluoridated toothpaste was not significantly associated with ECC or S-ESS, despite the fact that it was used by 70.7% of children; nor was demonstrated an associations with professional fluoride varnish application. Children in this survey were not receiving fluoride supplementation, unlike German children [41]. In a study by Kühnisch et al. [41], children receiving fluoride/vitamin D supplementation in the first year of their life had a significantly smaller chance of developing carious lesions in comparison to those supplemented for fewer than 6 months. Reported caries experience in Inuit preschool children significantly differed between those supplemented with vitamin D or fluoride and those not supplemented (75.5% vs. 60.0%, respectively, *p* < 0.01) [40].

The concentration of 25-hydroxyvitamin D [25(OH)D] in the blood provides a reliable indication of vitamin D levels [9, 10, 16–18, 38]. The first pilot studies conducted by Schroth et al. [16, 17] revealed that caries-free children were twice as likely to have optimal 25(OH)D concentrations ( ≥ 75 nmol/L ) and those with S-ECC had nearly three times higher chance of having deficient levels (< 35 nmol/L) [17]. Additionally, Wagner and Heinrich-Weltzien [32] concluded that children not supplemented with vitamin D had a 1.9 times higher probability of developing a carious lesion at the age of 3 years than those who were not. The present study clearly indicated that not using vitamin D supplementation, based on parental reports, should be regarded as a risk factor of caries development, similarly to other studies [12, 18, 21, 32]; thus, current caries risk assessment forms may need to be modified with inclusion of the vitamin D supplementation [32]. This study finding of significant association between parental-reported vitamin D supplementation and dt scores make it possible to formulate an initial thesis that higher supplementation may be protective against caries. The idea that vitamin D supplementation may be protective against caries is possible and supported by other studies [9, 12, 18, 21, 32, 38, 42]. It has been shown that 25(OH)D concentrations between 75 and 100 nmol/L offer protection against caries [9, 18, 42]. Also higher prenatal 25(OH)D levels may be protective [38]. According to the Institute of Medicine (IOM), adequate vitamin D level (≥50 nmol/L) ensures 47% lower odds for dental caries [9] and this finding is concluded from cross-sectional data based on a nationally representative sample, which gives it sufficient relevance. Similarly to Canadian populations, Polish children are also at risk of vitamin D deficiency due to relatively high latitude geographical location.

Studies assessing the association between vitamin D and caries have not always high research quality, or they presented contradictory results [5, 13–15, 43–45]. Some studies did not confirm such an association [5, 15, 44, 45]. A randomised study on English children did not support any strong association between 25(OH)D and caries prevalence and severity; however, it found only weak evidence for caries onset [5]. The authors of the National Health and Nutrition Examination Survey did not find a significant association between vitamin D and caries experience in US children (*p* = 0.78), even after adjusting for sugar consumption (*p* = 0.46) [44]. The present results failed to demonstrate the association in infants and children actually receiving vitamin D supplementation based on parental reports. In the case of infants, this may be due to the fact that almost all children received vitamin D in their first year of life. These children had fewer teeth than 3-year-olds, and so the time of presence of these teeth in the mouth was shorter, as well as the duration of performing inappropriate oral health-related practices. Also, Bucak et al. [15] concluded that the use of vitamin D during the first 6 months of life had no statistically significant association with ECC (*p* = 0.54).

Schroth et al. [16–18] were the first to show that prenatal 25(OH)D levels might have an influence on the primary dentition and the development of ECC. Specifically, maternal lower levels of vitamin D were associated with increased risk of dental caries in infants [14, 18, 19, 46].

The strengths and limitations must be taken into account. The strength of this study includes a large representative population at high-risk of low vitamin D status and ECC and homogenous social structure, without different cultures or ethnicities. There are no other similar Polish data. Another consideration is the use of both a questionnaire and a clinical examination for caries, and not reported-caries experience (RCE). Dental caries was determined by clinical evaluation and was scored by trained and calibrated, experienced paediatric dentists. The questionnaire findings were internally validated. The parental reports concerning caries could not be validated with the current data. Furthermore, since ECC has numerous causes, the study did not examine all of them, but focused on the factors with an important association. Naturally, this study was a cross-sectional study; therefore, has relatively low validation to draw conclusions regarding the causative association between parental-reported vitamin D supplementation and dental caries. In the future, longitudinal, prospective studies should be conducted to confirm categorically the beneficial association of vitamin D supplementation. In the present study, over-reporting is another limitation. Paediatricians provide parents or caregivers with instructions, and so the latter are aware of the necessity of supplementation. This is a potential risk of a response bias in the questionnaire reply, constituting a recognised limitation of parental-reported assessment in all such surveys. It is a fact that there are families that do not provide the supplementation consistently. The inability to validate vitamin D doses and regular intake, as well as the use of other supplements, was a significant limitation. The vitamin D supplementation was not categorised based on children’s age. Another factor that has to be considered is that the study group represented populations supplemented with vitamin D in the first year of life. Investigated 3-year-old children continued the supplementation during autumn–winter periods. The respondents were not asked specifically about supplementation in the second year of life, but about continuation. The authors of the present study encountered limitations due to a lack of information on several potentially important confounders, including information on seasonal variation, sun exposure, dietary intake of vitamin D-rich and fortified foods, prenatal vitamin D/multivitamin use. It may be explained, in line with surveys, by low UVB exposure (or “vitamin D winter”) lasting in Poland, and in similar geographic regions, and low intake of vitamin D–rich food [25–28, 35, 36, 42, 47]. Fortifying/enriching food with vitamin D has not been practised or widely available in Poland, with the exception of modified milk [26]. If a child drinks one litre of modified milk a day, the recommended vitamin D daily intake is sufficient [26]. With that assumption in mind, in the questionnaire there was only one question concerning milk. The identification of lower milk intake, however, proved irrelevant. On the other hand, it is true that vitamin D acquisition for foodstuffs can be particularly challenging for people who avoid the sun. Avoiding sunbathing and using of UV filters/blockers, environmental pollution, are implicated in the deficiency of vitamin D in children [25, 26]. For this reason, the present study has focused on the analysis of vitamin D supplementation. The inability to validate vitamin D intake and supplement use was a significant limitation. The questionnaire was not based strictly on the literature concerning vitamin D questionnaires (VDQ) to quantify the intake of vitamin D through diet and sun exposure and was un-validated. The input from researchers and clinicians was limited.

Nevertheless, it has to be mentioned as a limitation of the study that parental reports were not verified/confirmed by taking blood samples to measure 25(OH)D level since no attempt was made to perform these tests due to lack of consensus regarding the indications for measurement of 25(OH)D in Poland [25]. Moreover, “it is important to clarify that serum vitamin D does not change the major structure of teeth since this structure remains constant until some extrinsic factor causes its wear. Notwithstanding, apparently vitamin D prevents caries lesions through immune regulation, promoting microbial eradication with peptide activity” [19]. Vitamin D supplements could be taken in combination with minerals (i.e. calcium and phosphate), since neither vitamin D alone nor minerals alone have been shown to produce systemic benefits [48].

Therefore, in the interpretation of these findings, these limitations should be taken into account; however, they do not interfere with the statistical power as the large sample size provides sufficient statistical power, allowing greater confidence in these findings. The results should be interpreted with caution and evaluated in additional research with 25(OH)D measurement and information on getting vitamin D from food and sun exposure.

## Conclusions

The evidence from this cross-sectional study in favour of a high prevalence and intensity of dental caries experience was found in a nationally representative sample of 3-year-old Polish children. Parental-reported vitamin D intake was not compliant with recommendations for this age group. Dental caries was significantly lower in children with vitamin D supplementation in comparison with children not receiving such supplementation. An association between parental-reported supplementation and ECC and S-ECC was not significant after controlling for many confounding variables. There may be an association between vitamin D supplementation and lower caries in children living in a geographical region which is at risk of vitamin D deficiencies. Furthermore, the study confirmed that more children of mothers with higher education received vitamin D supplementation and had less caries. Maternal education, sweetened beverages before bedtime, bottle use were significantly associated with S-ECC. Greater emphasis should be placed on promoting vitamin D. Parents should supplement their children during periods of significant growth and development. Future longitudinal studies of such an association should be undertaken.
